# PathoSpotter-K: A computational tool for the automatic identification of glomerular lesions in histological images of kidneys

**DOI:** 10.1038/srep46769

**Published:** 2017-04-24

**Authors:** George O. Barros, Brenda Navarro, Angelo Duarte, Washington L. C. dos-Santos

**Affiliations:** 1 Universidade Estadual de Feira de Santana (UEFS) - Programa de Pos-Graduacao em Computacao Aplicada, Feira de Santana, Bahia, Brazil; 2 Fundacao Oswaldo Cruz (FIOCRUZ) - Instituto Gonçalo Moniz, Salvador, Bahia, Brazil

## Abstract

PathoSpotter is a computational system designed to assist pathologists in teaching about and researching kidney diseases. PathoSpotter-K is the version that was developed to detect nephrological lesions in digital images of kidneys. Here, we present the results obtained using the first version of PathoSpotter-K, which uses classical image processing and pattern recognition methods to detect proliferative glomerular lesions with an accuracy of 88.3 ± 3.6%. Such performance is only achieved by similar systems if they use images of cell in contexts that are much less complex than the glomerular structure. The results indicate that the approach can be applied to the development of systems designed to train pathology students and to assist pathologists in determining large-scale clinicopathological correlations in morphological research.

Pathology is a medical specialty that diagnoses diseases using the morphology of cell and tissue lesions observed in cytology smears and in histological slides. Cytology smears contain dispersed cells obtained from body secretions, aspiration biopsies, or scraped surfaces. Histological slides are tissue sections of 2–4 μm in thickness that are stained using techniques designed to highlight different structures^1^,^2^. Experienced pathologists can integrate clinical, laboratory and morphological data to generate an anatomopathological diagnosis, which supports the treatment and provision of prognosis of diseases. Although the anatomopathological diagnosis is complex, its morphological component is built upon elementary lesions associated with various diseases in a variety of ways^1^,^3^. For instance, glomerular hypercellularity (an elementary lesion) may be present in several types of kidney diseases, such as post-infectious glomerulonephritis, membranoproliferative glomerulonephritis associated with systemic lupus erythematous, or diabetic glomerulopathy^4^. Elementary lesions can be objectively defined and constitute the basis of the most precise and effective communication between pathologists regarding anatomopathological diagnosis criteria^3^. They also form the basis for teaching young pathologists.

Since the 1950s, several researchers have devoted their time to finding automatized classification techniques that monitor cell changes in cytology smears^5^. Although cytology classification systems still require further study, their use has contributed to decreasing the subjectivity and workload in cytology analysis^6^. Advances in cytology automation have stimulated developments in the analysis of tissue sections^7^. Cytology analysis mainly relies on the characteristics of dispersed or partially dispersed cells. The analysis of tissue sections relies on changes in cell morphology and the relationship between the cells and extracellular components. In the 1990s, advances in radiological digital imaging enabled the digital analysis of biomedical structures^8^. Hence, automated or semi-automated computer-assisted measurements have become common practice in pathology. Most of the development in the computational analysis of biological tissue imaging in pathology has occurred for cancer classification, where cell changes are predominant^9^. These developments are usually an extension of the process that extracts cell chromatin characteristics, as used in cytology, or analyses of the color and texture of images obtained from tissue sections stained via immunohistochemistry. Little development has occurred for the histological analysis of non-neoplastic lesions; cell changes in this case may be subtle, while the cell–extracellular matrix relationship is increasingly relevant. There are even fewer advances with respect to nephrological diseases, for which the diagnosis relies on multiple elementary lesions and strong clinical-pathological correlations^10^.

The translation of elementary nephrological lesions into some form of computational language would enable large-scale clinical-pathological associations via a large database and contribute to the training of young pathologists. In this study, we present the early results related to the ability of PathoSpotter-K to identify elementary glomerular lesions. PathoSpotter-K is a branch of the PathoSpotter project, which is an interdisciplinary research project that joins pathologists and computer scientists with the aim of generating computational systems that support research and training in pathology.

## Materials and Methods

**Figure 1:**
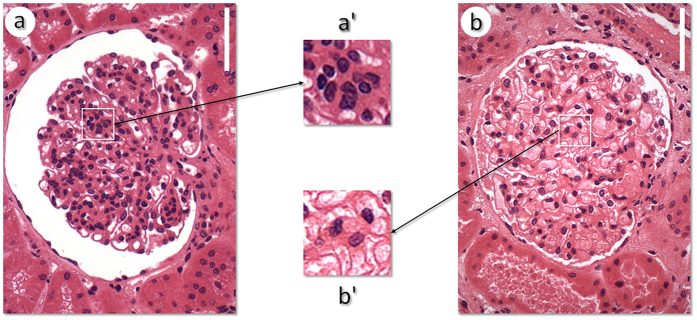
Two images of glomeruli. (**a**) Glomerulus with a proliferative glomerulopathy. (**b**) Normal glomerulus. The enlarged areas (**a**’,**b**’) emphasize cell clusters and highlight proliferative vs non-proliferative areas, respectively. Stained with hematoxylin and eosin. Magnification bar = 60 μm.

The standard method used to diagnose glomerular hypercellularity is to visually inspect histological sections from the glomeruli, searching for the presence of clusters formed by four or more cell nuclei in the mesangial area or by cell aggregates that fill the capillary lumen^1^,^3^; this change is the key to distinguishing proliferative glomerulopathy. As an example, in Fig. 1a, the enlarged circle highlights a cell cluster in the mesangial area and a glomerular capillary lumen filled with nucleated cells in the case of proliferative glomerulonephritis. Figure 1b illustrates a normal glomerulus, with no significant clusters in the mesangial area or in the capillary lumen. In both figures, the nuclei are distinguished as structures with a strong dark blue color.

There are no standard or robust criteria for nuclear segmentation that would allow for defining a cluster of nuclei; therefore, the identification of clusters of nuclei is subjective and highly dependent on a pathologist’s experience. This subjectivity makes standardization of this method via computational techniques particularly challenging to implement because modeling the expertise of a pathologist is very difficult^11^. Therefore, as in any conventional pattern recognition system, our first task was to identify a set of attributes (features) from the images to build a feature vector that effectively differentiates normal glomeruli from glomeruli with cell proliferation. After performing preliminary analyses and experiments, we realized that the ratio between the number of nuclei and the number of clusters could be a good discriminant feature. At this point, we focused on finding a way to automatically extract this ratio to perform automatic image classification. The next sections describe the methods used to achieve this goal. PathoSpotter-K was developed with Python 2.7 using algorithms from the Scikit-image library (image processing functions) and from the Scikit-learn library (machine learning functions)^12^,^13^.

### Renal biopsy

Small kidney fragments were obtained either by percutaneous needle puncture or surgically and processed according to the recommended guidelines^2^. Briefly, the biopsies were fixed in formalin (to preserve their histological structure), embedded in paraffin, cut into 2–3 μm thick sections, and finally stained using one of the following techniques: hematoxylin and eosin (H&E) and periodic acid–Schiff (PAS), periodic acid-methenamine silver stain (PAMS), Mallory’s trichrome (AZAN) or picrosirius red stain. The images used in this study were from renal tissue sections stained with H&E and PAS according to the following protocol:

For H&E staining, the sections of renal tissue were immersed for 1 minute in Harris’ hematoxylin, rinsed for 5 minutes under running water, dipped in 1% eosin for 30 seconds and placed in a 1% acetic acid solution for 10 seconds. For PAS staining, the kidney tissue sections were dipped in 1% periodic acid for 10 minutes, washed under running water and submerged in Schiff’s reagent for 30 minutes. They were washed again for 2 minutes under running water, dipped in Harris’ hematoxylin for 1 minute and washed under running water for 5 minutes. After staining, the sections were dehydrated in alcohol, clarified in xylol and mounted using Canada balsam.

### Digital image library

The digital image library was built by Dr. Washington LC dos-Santos (MD) and includes images of all the kidney biopsies performed for the diagnosis of glomerular diseases in referral nephrology services of the public hospitals of Bahia State, Brazil; the images were examined at the Gonçalo Moniz Institute – Fiocruz (IGM-FIOCRUZ) between 2003 and 2015. The image library contains approximately 80,000 digital images of various types of renal disease. Only slides with high technical staining quality were used for producing the images included in the library. The general characteristics of the patients whose renal biopsies were used to produce the image library are shown in Table 1.

### Data set

**Figure 2:**
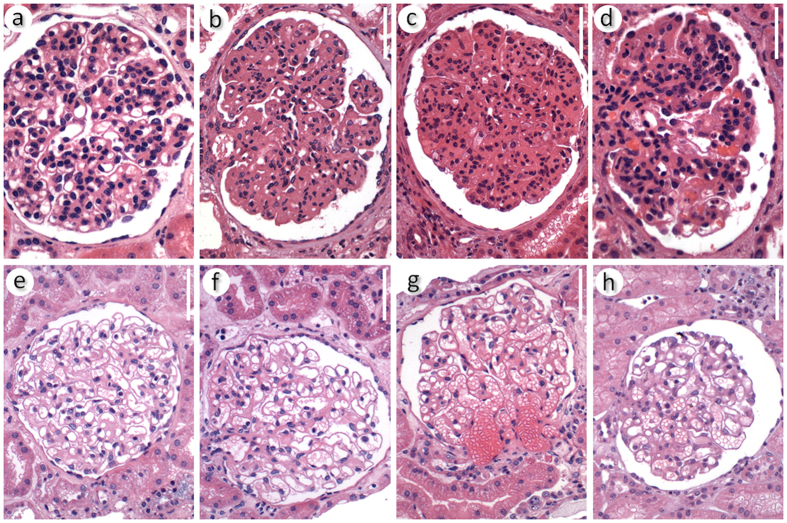
Representative images from the dataset. Images (**a–d**) represent glomeruli with proliferative lesions. Images (**e–h**) represent normal glomeruli. Stained with hematoxylin and eosin. Magnification bar = 60 μm.

The digital images of glomeruli used in this work were selected from the digital image library described above. They were captured using an Olympus QColor 3 digital camera attached to a Nikon E600 optical microscope (using x200 magnification). The dataset images differed based on various features, such as the type of dye used to stain the tissue sections (either H&E or PAS) and morphological differences inherent to a patient’s age, type of disease, glomerulus position, and section plane. In this work, we used 811 images, including 300 images of normal glomeruli and 511 images of glomeruli from kidneys with proliferative glomerulopathies. Some of these images are presented in Fig. 2.

### System Architecture

**Figure 3:**
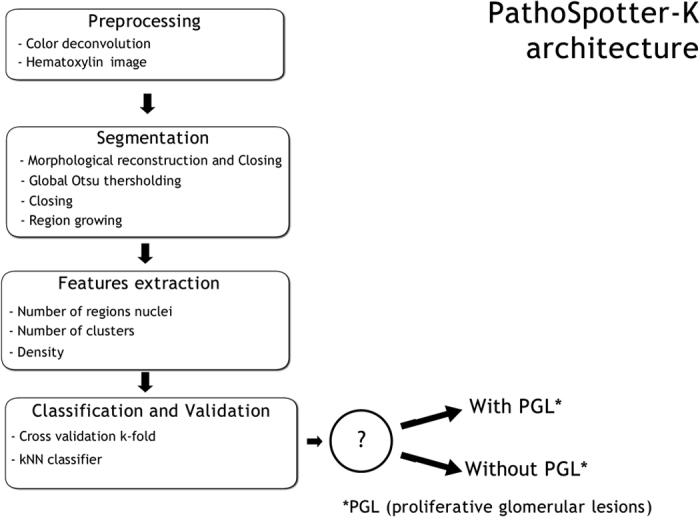
PathoSpotter-K system architecture.

As shown in Fig. 3, PathoSpotter-K follows the typical architecture of a conventional image pattern recognition system^14^. The system consists of four sequential stages: preprocessing, segmentation, feature extraction and classification.

### Preprocessing

In this stage, two operations are performed: the areas of interest are highlighted, and noise is reduced. The first operation changes the images’ color space to facilitate the segmentation process to be performed in the next stage. Such changes were made using the color deconvolution method proposed by Ruifrok and Johnston, which was successfully used as a digital pathology method that segments different types of structures in biological tissues^15^,^16^,^17^. The color deconvolution is an image-processing algorithm that provides a robust and flexible method for representing a glomerulus image in channels, separating the dyes that were used to acquire it. The image processing library Scikit-image, developed by Van der Walt and colleagues^12^, contains a method for converting the color space from RGB to HED. The HED color space stores the information from hematoxylin, eosin, and DAB as three separate images. We chose the hematoxylin color channel, represented by a matrix **H** (in which each element is in the range [0, 255]), herein referred to as the H-matrix, because the nuclei were stained using hematoxylin and thus yield the brightness information in the **H**-matrix. Consequentially, the H-matrix can be interpreted as an image containing only the areas in the original image that are stained by hematoxylin.

The H-image is contaminated by noise related to textural information, which complicates the segmentation process before the feature extraction. For instance, nuclei fragments may produce noise. The nuclei fragments interfere in the segmentation phase because a fragment may be misclassified as a whole nucleus. To minimize this interference without having to tackle an increase in the computational complexity to differentiate the fragments, we treated the nuclei fragments that are large enough to pass through the noise reduction filter as whole nuclei.

**Figure 4:**
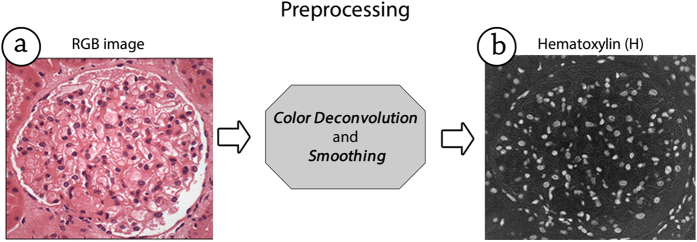
Operation for separating the hematoxylin information (**b**) from the original colored image (**a**).

To reduce such noise, we applied a smoothing filter based on the median filter, with a window of 3 × 3 pixels, which eliminates the noise interference while preserving the borders of the nuclei, which is crucial for accurate segmentation. Figure 4 depicts the operations result of the preprocessing stage. Since we transformed the images to black and white mode before segmenting the nuclei, and since the color separation method used works regardless whether sections were stained using the H&E or PAS technique, PathoSpotter-K is not sensitive to the dye at the moment.

### Segmentation

The segmentation method used to separate the nuclei in the image was inspired by the work of Miranda and colleagues (2012), who isolated structures from cervical cell images; the work of Mathur and colleagues (2013), who isolated structures from blood white cell images; and the work of Schochlin and colleagues, who isolated structures from skin cancer cell images^18^,^19^,^20^.

**Figure 5:**
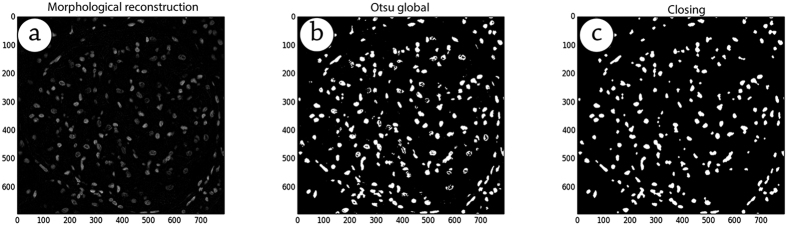
Segmentation stages.

We used the morphological operations of closing for reconstruction, together with the Otsu method to segment the cell nuclei. Initially, we performed a closing morphological operation using the top-hat operator to highlight the cell nuclei in the images. After using the closing morphological operation, we applied the Otsu method to generate binary images (only black and white pixels). Finally, we performed the closing morphological operation again to improve the representation of the nuclei and to reduce the noise introduced by the binarization. Figure 5 shows the steps performed in the segmentation stage.

The image produced via the Otsu method is a binary image that depicts nuclei in white and everything else in black. After binarization, we used the method of growing by pixel-aggregation to identify nuclei and clusters. This method begins by searching for pixel-seeds that satisfy specific properties. These seeds serve as starting points to identify surrounding pixels to build a region. After identifying a seed pixel (we used the pixel brightness as a property), the area around this pixel grows by including neighboring pixels with similar properties until there are no pixels left to attach. Once the region is created, it receives a unique identifier, and the algorithm searches for other regions in the image. The identification of regions via edge and neighborhood analysis was performed using the “regionprops” method from the Scikit-image library. The resulting array contained information specific to each identified region.

### Feature Extraction

This stage extracts quantitative information required to differentiate images with or without proliferative glomerular lesions. We tested three image metrics as candidates for discriminants: 1) the number of regions with nuclei, 2) the agglomeration of nuclei and 3) the pixel density. These metrics were correlated with the proliferation that occurs in a glomerulus resulting from proliferative lesions. The number of regions with nuclei was counted using the “regionprops” property in the Scikit-image library, which uses edge detection and region growth methods. Each region was characterized by a group of pixels with the same brightness. The number of agglomerations was extracted using the Laplacian of Gaussian (LoG) method – also implemented from the library – which works as a bubble detection method. The LoG method was used to identify the number of nuclei from neighboring cells that were close enough, but not necessarily bound to each other, to form a cluster. Finally, the last characteristic, pixel density, was computed by dividing the number of white pixels (pxW) by the number of black pixels (pxB).

**Figure 6:**
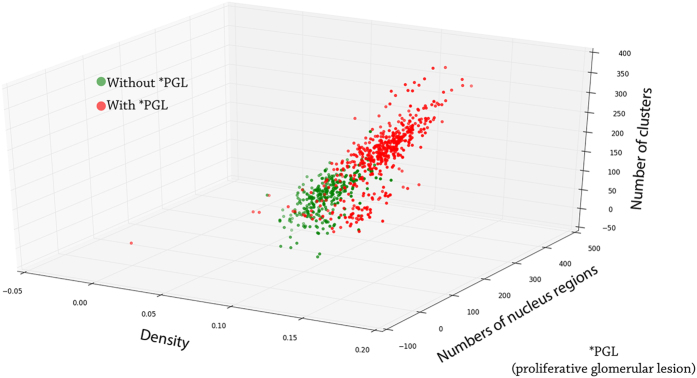
Distribution of the images into the feature space.

To analyze if the metrics were sufficiently able to indicate a lesion, we compared them using histograms. Each histogram plots the distribution of images for each specific metric. A good metric must clearly differentiate the images of normal glomeruli from the images of lesioned glomeruli. Figure 6 shows the distribution of the images relative to the combination of metrics.

### Classification and Validation

**Figure 7:**
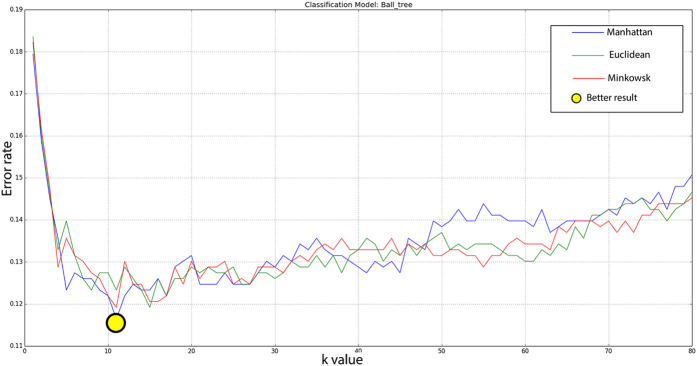
Error rates for the different kNN models tested. The yellow dot indicates the better configuration (Manhattan with k = 11).

**Figure 8:**
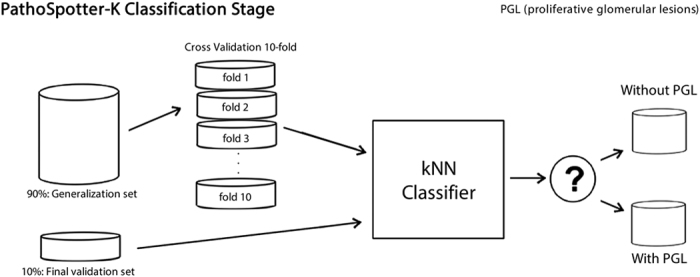
Organization of the datasets used to configure the kNN classifier. The generalization set yielded 10 different subsets according to the k-fold method.

PathoSpotter-K uses a binary classifier based on the kNN (k-Nearest Neighbor) algorithm. The classifier was adjusted using 90% of the images from the dataset as the generalization set. The remaining 10% was separated for assessing the classifier performance (validation set). We used the k-fold cross validation method to split the generalization set into 10 subsets to parametrize the classifier. This number of subsets was chosen based on the literature^21^,^22^,^23^. To parameterize the kNN classifier, we adjusted the distance metrics and the k-parameter (nearest neighbors). We also tested the Euclidean, Manhattan and Minkowski distance metrics with values of k ranging from 3 to 80. The maximum value of k corresponded to the total number of samples in each fold, and the minimum values corresponded to the number of classes plus 1. We evaluated the classifier’s model and the parametrization by assessing the error rate^24^. Figure 7 depicts the relation between the three distances and the k-parameter. The yellow dot indicates the configuration for the lowest error, which corresponds to the Manhattan distance using k = 11. The precision, recall, accuracy and standard deviation were obtained using the scheme depicted in Fig. 8.

### Ethical considerations

The study was conducted in accordance with resolution No. 466/12 of the National Health Council. To preserve confidentiality, the images (including those shown in the paper) were separated from the other patient data. No data presented herein allows patient identification. All the procedures were approved by the Ethics Committee for Research Involving Human Subjects of the Gonçalo Moniz Institute from the Oswald Cruz Foundation (CPqGM/FIOCRUZ), Protocols No. 188/09 and No. 1.817.574.

## Results and Discussion

The classifier’s performance was evaluated using the metrics derived from the confusion matrix^25^. Using the generalization set, the system yielded 92.3% precision, 88.0% recall and 88.3 ± 3.6% accuracy. Using the validation set, we obtained 88% precision, 88% recall and 85% accuracy. Compared to similar work in the digital pathology literature, the 88% accuracy achieved by PathoSpotter-K is equivalent to the results obtained by Kothari and colleagues (77%)^26^, Sirinukunwattana and colleagues (77%)^23^, Schochlin and colleagues (88.9%)^20^, and Mathur and colleagues (92%)^18^.

Although the results of PathoSpotter-K can be compared to similar works performed using cytological smears and images of cell changes that are much less complex than images from histological sections, we believe that the imbalance between the number of glomeruli images with and without lesions has mostly influenced the parametrization and, as a consequence, the final performance of the classification. The reason for this imbalance is that the original dataset images were obtained with the intention to teach others regarding the diagnosis of kidney diseases and not to build a classification system. Currently, this dataset is being populated with images of normal glomeruli so that we can reparametrize the system to improve its performance in the near future.

## Concluding Remarks

In this paper, we presented PathoSpotter-K, a computational system that assists pathologists in teaching and researching nephrology using digital images of glomeruli. We used a combination of classical image processing and machine learning methods to create a pattern classification system for kidney pathology, an understudied area in digital pathology. A web service is under construction so that we can simplify the access to our system and increase the number of pathologists and computational science professionals who can help to improve the system. We intend on improving the accuracy of PathoSpotter-K by searching for better image metrics and using additional classification algorithms. We will also increase the number of samples in the dataset to better parametrize the data while improving robustness. Additionally, we want to expand the capabilities of PathoSpotter-K to classify additional classes of glomerular lesions.

## Additional Information

**How to cite this article:** Barros, G. O. *et al*. PathoSpotter-K: A computational tool for the automatic identification of glomerular lesions in histological images of kidneys. *Sci. Rep.* **7**, 46769; doi: 10.1038/srep46769 (2017).

**Publisher's note:** Springer Nature remains neutral with regard to jurisdictional claims in published maps and institutional affiliations.

## Figures and Tables

**Table 1:** General characteristics of the patients submitted to renal biopsies for diagnostic purposes and used to produce the image library.

Parameter	Cell Proliferation		Total
	Yes	No	
N	869 (100%)	439 (100%)	1308 (100%)
Sex:			
Female	486 (56%)	203 (46%)	689 (53%)
Male	382 (44%)	236 (54%)	618 (47%)
Age	29 ± 16	31 ± 17	30 ± 16
Most frequent diseases:			
Focal and segmental glomerulosclerosis	167 (19%)	161 (37%)	328 (25%)
Lupus nephritis	255 (29%)	33 (8%)	288 (22%)
Membranous glomerulonephritis	63 (7%)	52 (12%)	115 (9%¨)
Membranoproliferative glomerulonephritis	80 (9%)	0 (0%)	80 (6%)
Minimal change disease	24 (3%)	56 (13%)	80 (6%)
Diffuse proliferative glomerulonephritis	68 (8%)	2 (1%)	70 (5%)
IgA nephropathy	45 (5%)	14 (3%)	59 (4%)
